# A multi-frame network model for predicting seizure based on sEEG and iEEG data

**DOI:** 10.3389/fncom.2022.1059565

**Published:** 2022-11-14

**Authors:** Liangfu Lu, Feng Zhang, Yubo Wu, Songnan Ma, Xin Zhang, Guangjian Ni

**Affiliations:** ^1^Academy of Medical Engineering and Translational Medicine, Tianjin University, Tianjin, China; ^2^School of Mathematics, Tianjin University, Tianjin, China; ^3^Tianjin Key Laboratory of Brain Science and Neuroengineering, Tianjin University, Tianjin, China; ^4^Laboratory of Neural Engineering and Rehabilitation, Department of Biomedical Engineering, College of Precision Instruments and Optoelectronics Engineering, Tianjin University, Tianjin, China; ^5^Tianjin International Joint Research Center for Neural Engineering, Academy of Medical Engineering and Translational Medicine, Tianjin University, Tianjin, China

**Keywords:** deep learning, EEG, multi-frame network, seizure prediction, feature extraction, pre-ictal

## Abstract

**Introduction:**

Analysis and prediction of seizures by processing the EEG signals could assist doctors in accurate diagnosis and improve the quality of the patient's life with epilepsy. Nowadays, seizure prediction models based on deep learning have become one of the most popular topics in seizure studies, and many models have been presented. However, the prediction results are strongly related to the various complicated pre-processing strategies of models, and cannot be directly applied to raw data in real-time applications. Moreover, due to the inherent deficiencies in single-frame models and the non-stationary nature of EEG signals, the generalization ability of the existing model frameworks is generally poor.

**Methods:**

Therefore, we proposed an end-to-end seizure prediction model in this paper, where we designed a multi-frame network for automatic feature extraction and classification. Instance and sequence-based frames are proposed in our approach, which can help us simultaneously extract features of different modes for further classification. Moreover, complicated pre-processing steps are not included in our model, and the novel frames can be directly applied to the raw data. It should be noted that the approaches proposed in the paper can be easily used as the general model which has been validated and compared with existing model frames.

**Results:**

The experimental results showed that the multi-frame network proposed in this paper was superior to the existing model frame in accuracy, sensitivity, specificity, F1-score, and AUC in the classification performance of EEG signals.

**Discussion:**

Our results provided a new research idea for this field. Researchers can further integrate the idea of the multi-frame network into the state-of-the-art single-frame seizure prediction models and then achieve better results.

## 1. Introduction

Epilepsy is a neurological disease characterized by recurrent seizures, repeats long or short severe convulsions, which may cause physical injury or even fracture (Wirrell, 2006). According to the statistics of the World Health Organization, there are about 50 million patients with epilepsy in the world (Carney et al., 2011). Among them, about 70% of patients with can be controlled by medical means such as drugs, but the seizures of the remaining 30% of patients with epilepsy can be controlled by anti-drug (Gadhoumi et al., 2016). For anti-drug seizures, surgical resection of the epileptogenic area or neural stimulation usually is considered by doctors to cure it. The methods based on neural stimulation promote the studies of seizure prediction models. However, one reliable seizure prediction model can be utilized to improve the quality of life of patients with anti-drug seizure, so that they can take safety measures and electrical stimulation in advance before a seizure, which can prevent serious adverse consequences (Freestone et al., 2017).

As we all know, the seizure is usually caused by abnormal brain activity, and the analysis of an EEG signal is a powerful means to discover the brain patterns (Litt et al., 2001). The traditional method of recording EEG signals is placing the electrode on the surface of the head, and the obtained signal is called scalp EEG (sEEG) (Rasheed et al., 2020). With the further development of medical technology, the electrode is implanted into the brain through minimally invasive methods. The EEG signal obtained in this way is called intracranial EEG (iEEG) (Lachaux et al., 2003). Compared with sEEG, the electrical activities of the cerebral cortex can be directly recorded by iEEG, which can avoid the influence of transmission media, such as scalp and cerebrospinal fluid, and the interference of artifacts, such as ECG and body movements. iEEG has a high signal-to-noise ratio which can intuitively reflect the symptoms of the seizure.

Another main feature of EEG is the relatively low hardware cost, which can be used to process a large scale data of huge number of patients and record EEG signals for a long time. Neurologists usually study EEG signals recorded for several days, weeks, or even several months to analyze seizure symptoms, which require a lot of human labor and time. Therefore, the seizure prediction model based on EEG data has always been a hot research topic.

The recent research on EEG-based seizure prediction originated in the 1970s (Mormann et al., 2007). Early researchers used linear methods, such as autoregressive analysis (Rogowski et al., 1981), to extract features that can predict seizures from EEG signals. In the 1980s, with the development of nonlinear methods, researchers utilized nonlinear analysis to do seizure prediction for feature extraction and achieved some improved results (Iasemidis et al., 1990; Martinerie et al., 1998; Le Van Quyen et al., 1999). In addition to linear and nonlinear methods, a variety of univariate and multivariate features have been proposed by researchers during this period, and Zhang et al. (2018) have made a relatively good summary of these features.

Though the state-of-the-art methods mentioned above have achieved good results in seizure prediction, they are not easy to be generalized (Mormann et al., 2007). In recent years, with the development of data science and big data technology, the acquisition of large EEG data sets has turned to be easier. Many researchers proposed seizure prediction models based on machine learning and deep learning methods which can be applied to large scale datasets, such as CHB-MIT (Shoeb, 2009) and Kaggle datasets (Brinkmann et al., 2016), and have achieved better results than the traditional ones. However, the framework of the model is relatively single and cannot deal with complex and dynamic data (Ung et al., 2017). Therefore, we proposed a seizure prediction model based on a multi-frame network in this paper, which aims to solve the above problems, and we designed ablation experiments to verify the effectiveness of the model as well.

The contributions of this paper are listed as follows. First, we proposed an efficient end-to-end seizure prediction model, which has no complicated preprocessing steps and can be directly used for raw data. Second, we designed a multi-frame network for automatic feature extraction and classification. The network contains instance-based and sequence-based frames, which can simultaneously extract features of different modes for further classification. Finally, we conducted experiments on two kinds of EEG datasets, namely the Kaggle dataset (iEEG) and the CHB-MIT dataset (sEEG), to demonstrate the generalizability of our model. A multi-frame network is proposed and validated in this paper, which can extract more effective embeddings even without preprocessing methods. In addition, we also conducted some comparisons on the existing models with similar applications to show the effectiveness of our approaches. It is worth noting that the presented method by combining two basic model architectures is generally compatible with most of the state-of-the-art single-frame seizure prediction frames, which can be used to boost the performance of these methods to achieve better results.

The structure of the rest of the paper is organized as follows. Section 2 briefly reviews the relevant background and research works. Section 3 introduces the main model frame multi-frame network in detail. Section 4 introduces the datasets, shows the evaluation methods, and reports the experimental results. Section 5 compares the model frame proposed in this paper with the existing model frame and discusses the experimental results. Finally, Section 6 draws the conclusions and puts forward the future research directions.

## 2. Related work

**Figure 1:**
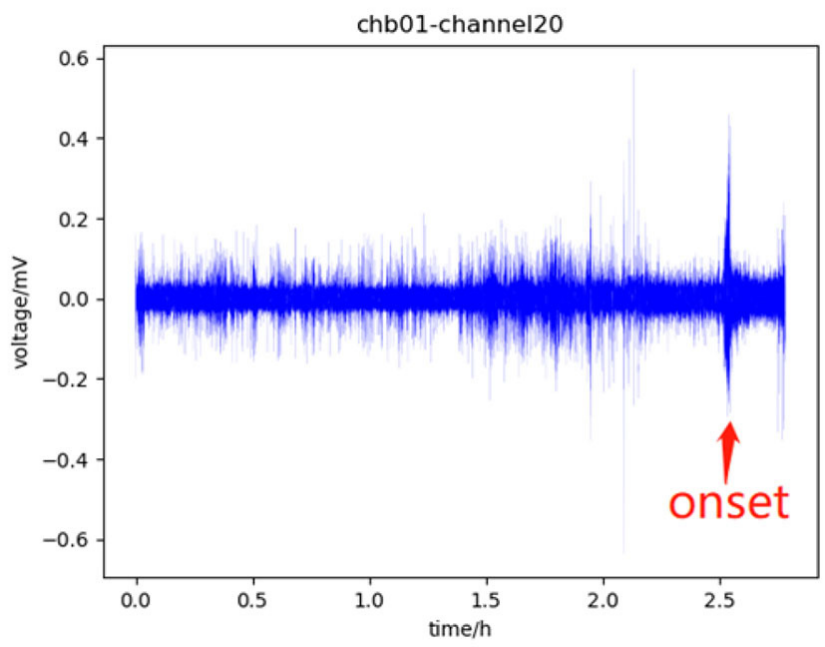
Part of the data of channel 20 of chb01 in the CHB-MIT dataset, in which 0–1.5 h is the inter-ictal, 1.5–2.5 h is the pre-ictal, there is a seizure onset near 2.5 h which is marked with a red arrow, which will last for tens of seconds, and the subsequent period is the post-ictal.

According to EEG signals, the human brain can be divided into four states: pre-ictal, which refers to a period before a seizure and usually lasts for tens of minutes; ictal, which refers to the period from the onset to the offset of the seizure; post-ictal, which refers to a period after the end of the seizure; the rest is called inter-ictal (Ullah et al., 2018). Before the onset of the seizure, the EEG signal in the pre-ictal will change slightly compared with the signal in the inter-ictal. This change indicates that the seizure will occur soon, which can assist doctors to make timely interventions to minimize the impact of the seizure episode. Therefore, in the study of the seizure prediction model, identifying pre-ictal from EEG signal is the main task, especially identifying pre-ictal from inter-ictal. Figure 1 shows the four states.

The seizure prediction models can be divided into two categories. The first is the traditional method, which extracts the features related to pre-ictal through complex feature engineering and then determines an appropriate threshold. When the features are lower than the threshold, the seizure will occur (Iasemidis et al., 2005). For example, Schelter et al. (2006) used the dynamic similarity index to predict seizure. The core of traditional methods is feature extraction. Features can be divided into time-domain features, frequency-domain features, time-frequency features, and nonlinear features (Yang et al., 2018). There are also studies that combine feature extraction with anomaly detection and stochastic processes to solve this problem (Fujiwara et al., 2015). However, these features do not take the individual differences between patients into consideration, and whether these features are applicable to all patients or not is still unclear. Moreover, due to the non-stationary characteristics of EEG signals, the traditional methods suffer from poor generalization and demonstrate performance close to random (Rasheed et al., 2020). The second category is based on machine learning and deep learning. The core task is the classification of inter-ictal and pre-ictal. Specifically, the feature space is obtained by extracting predefined features. After appropriate transformation of the feature space, the classifier is trained by labeled data and then will be used to classify pre-ictal and inter-ictal. The large datasets, such as CHB-MIT and Kaggle datasets, are very contributive to the training of the model. Therefore, the seizure prediction model based on machine learning and deep learning is the focus of seizure prediction research nowadays.

The advantages of machine learning are lightweight and artificially specified features, which are interpretative and can be implemented quickly so as to be deployed to devices (Cook et al., 2013; Teijeiro et al., 2019). For example, Messaoud and Chavez (2021) extracted 24 features from CHB-MIT and Kaggle datasets, input the reduced features into a random forest classifier, and achieved good results. Yuan et al. (2018) proposed a novel feature named diffusion distance, and Bayesian linear discriminant analysis was used for classification. Anandaraj and Alphonse (2022) incorporated the feature extraction phase and feature selection phase to enhance the generalization capability and input the features to a boosted ensemble model for training and prediction. In addition, logistic regression, support vector machine, and k-nearest neighbor are also commonly used in classification. A review showed that random forest is the best classifier in these methods (Lekshmy et al., 2022). However, due to the wide variety of features that can be extracted, sometimes additional feature selection methods were needed to improve the efficiency of feature extraction (Wang and Lyu, 2014).

The advantage of deep learning is to automatically extract appropriate features (Abdelhameed and Bayoumi, 2021; Li et al., 2022; Xu et al., 2022), saving the work of selected features for specific patients or specific times. Acharya et al. used a convolutional neural network (CNN) to analyze EEG signals for the first time (Acharya et al., 2018). He preprocessed the initial EEG signals, converted them into a format with a mean of 0 and a standard deviation of 1, and then input them into CNN to obtain the final classification results. The most common preprocessing method is time-frequency analysis, such as fast fourier transform (FFT), short time fourier transform (STFT), and wavelet transform (WT). The EEG signals are transformed into spectrums and then are fed into the network. For example, the multi-view CNN (Liu et al., 2019) proposed by C. L. Liu et al. obtained the time-domain features and frequency-domain features after FFT and principal component analysis of EEG signals, respectively, and used them as the input of the model. In addition, Truong et al. (2018) obtained the spectrum of EEG signal through STFT as the input of CNN, which also achieved good results. Khan et al. (2017) obtained the spectrum of EEG signal through WT as the input of CNN.

In addition to the CNN-based models, there are also recurrent neural network-based models, which extracted the time dependency in EEG signals. K. M. Tsiouris et al. first used Long Short-Term Memory deep learning network (LSTM) to predict seizures (Tsiouris et al., 2018). After extracting EEG signals as feature vectors, he used LSTM to classify sequences consisting of feature vectors and achieved the expected performance. Singh and Malhotra (2022) proposed a spectral feature-based two-layer LSTM network model for the automatic prediction of epileptic seizures using long-term multi-channel EEG signals. Moreover, LSTM is sometimes used in conjunction with CNN. For example, Shahbazi and Aghajan (2018) proposed a CNN-LSTM architecture, which captures the time-frequency features using CNN first and then captures temporal patterns using LSTM second. There are some models based on transfer learning that have similar architectures. Abdelhameed and Bayoumi (2018) proposed a semi-supervised seizure prediction model based on CNN self-encoder, which reduces the dimension and compresses the original EEG data in an unsupervised way and then trains an LSTM classifier with supervised learning. They both first use CNN to extract features and then use LSTM to extract features. The two steps are not simultaneous.

The seizure prediction models mentioned above showed that the existing methods relied heavily on complex feature extraction or preprocessing methods and were based on either CNN or RNN. Despite there being CNN-LSTM networks, however, its feature extraction is not simultaneous and needs to be in a certain order. The framework of the above model is relatively single. A single-frame model usually transforms the raw data into a low dimensional feature mapping, which may lead to the excessive dimensionality reduction of data after the first feature extraction using CNN and affect the RNN's secondary feature extraction or classification. In seizure prediction applications, it is necessary to develop a new model framework to extract more discriminative features. Therefore, this paper proposed a seizure prediction model based on multi-frame network to solve the above problems and provided a new research idea in this field.

## 3. Methodology

**Figure 2:**
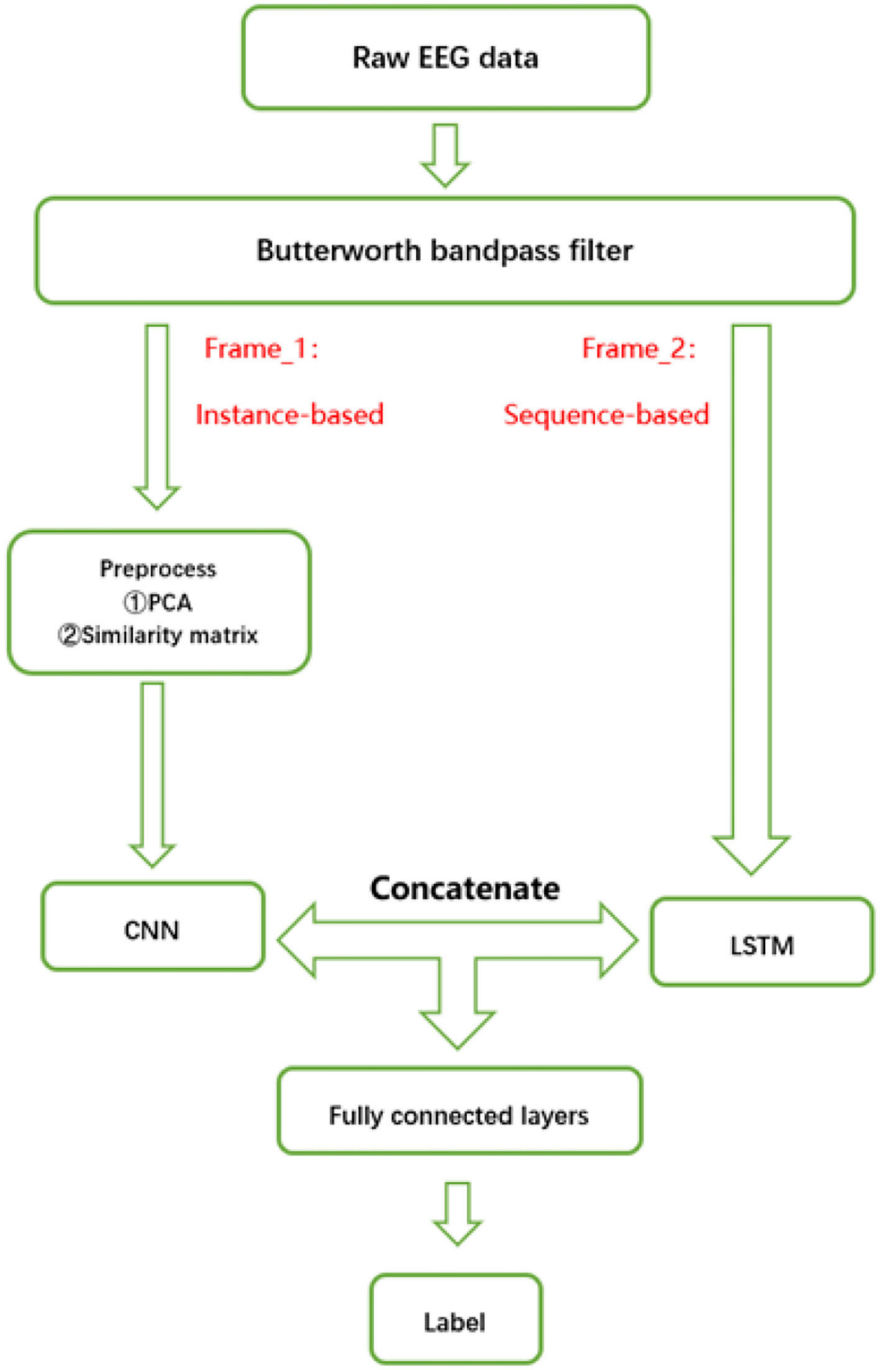
Structure of the multi-frame seizure prediction model.

In this paper, the seizure prediction model based on a multi-frame network mainly included two parts: preprocess and classification. The framework of the whole model is shown in Figure 2. The specific implementation will be introduced step by step in this section.

### 3.1. Data preprocessing

To separate the useful signal from the noise and improve the signal-to-noise ratio of the signal, we need to filter out the noise and artifacts. This step can be completed by using a Butterworth bandpass filter to filter the original signal (Robertson and Dowling, 2003). Then, the obtained signal is further divided into non-overlapping segments with a duration of 1 s. The size of each segment is (N, M), where N represents the number of channels of the subject and M is the frequency.

To extract the spatial information of segments, we need to transform the original segments, which can also reduce the dimension of the data and model complexity. For the segment with the size of (N, M), we use principal component analysis (Abdi and Williams, 2010) to extract the first N principal components to transform it into a matrix with the size of (N, N). In the matrix, each row represents an electrode, and each column represents a principal component.

**Figure 3:**
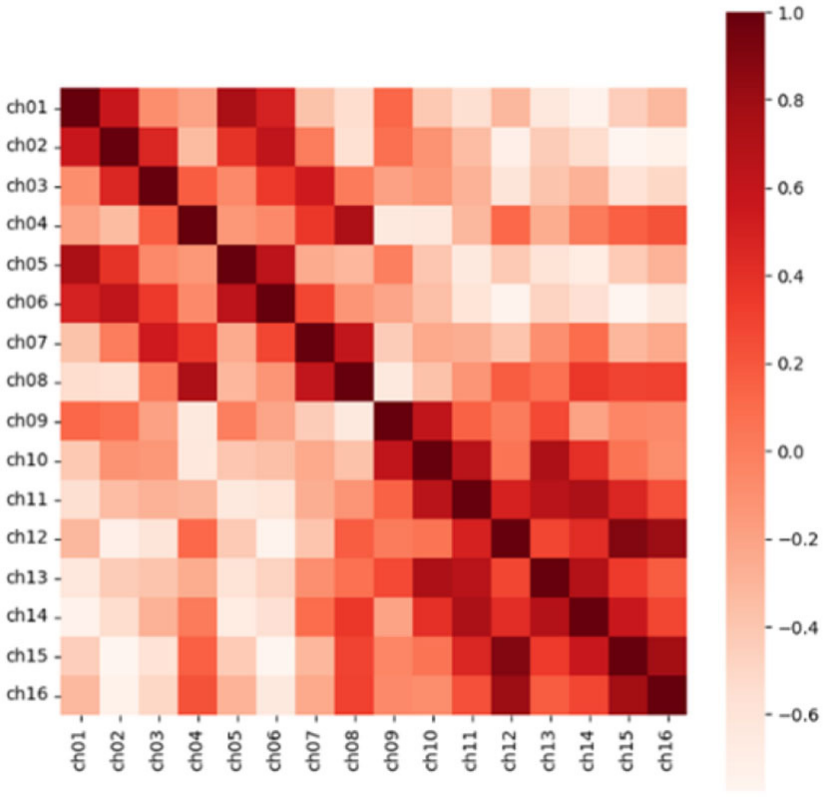
The heat map of the correlation coefficient matrix extracts the position information through the correlation between the electrodes. Source data is from the CHB-MIT dataset.

In addition, for N channels, we calculated the Pearson correlation coefficient in pairs to obtain the similarity matrix R with the size of (N, N), which was to consider the correlation between electrodes in feature extraction. Taking a segment of Dog_1 in the Kaggle dataset as an example, the similarity matrix we obtained is shown in Figure 3. The Pearson correlation coefficient *r*_*xy*_ of channel x and channel y is calculated as follows.


(1) $$\begin{array}{l} r_{xy}=\frac{n\sum x_{i}y_{i}-\sum x_{i}\sum y_{i}}{\sqrt{n\sum x_{i}^{2}-{\left(\sum x_{i}\right)}^{2}}\sqrt{n\sum y_{i}^{2}-{\left(\sum y_{i}\right)}^{2}}} \end{array}$$

Finally, the segment with a size of (N, M) will be transformed into a third-order tensor with a size of (2, N, N). It is worth noting that usually the number of segments in the pre-ictal period is much less than that in the inter-ictal period, thus, the two types of segments in the dataset are imbalanced. As a result of imbalanced training data, it will affect the training of the model, we randomly selected the same number of inter-ictal segments as that in the pre-ictal period, which ensured that the above two types of data are balanced.

### 3.2. Multi-frame network classification

In previous studies, some researchers regarded the signal as an instance and used a CNN to extract spatial features. On the other hand, other researchers regarded the signal as a time series to use an RNN to extract sequence features. Therefore, the signal features can be extracted from two frames: instance-based or sequence-based. However, to the best of our knowledge, there is no model to extract two types of features simultaneously. Although there are some models similar to CNN-LSTM (Abdelhameed and Bayoumi, 2018; Shahbazi and Aghajan, 2018) using CNN first and then LSTM to extract features, due to the deep configuration network, there is the possibility of losing information in the process of propagation.

For the above reasons, for each segment, we extracted instance-based and sequence-based features simultaneously through two sub networks in parallel and then concatenated the two features to obtain a high-level representation of the segment.

#### 3.2.1. Instance-based feature detection

LeNet was first proposed by LeCun et al. in 1989 to recognize handwritten digits in images (LeCun et al., 1998). Later, AlexNet proposed by Krizhevsky et al. (2012) made a great improvement in the 2012 ImageNet challenge, and CNN began to be widely used in the field of computer vision. CNN can be used to extract high-order spatial features and has relatively few parameters.

To extract the spatial information of a segment, in preprocess period, a segment with a size of (N, M) was transformed into a third-order tensor with a size of (2, N, N), which is beneficial for CNN to extract spatial features. This is because the convolution kernel of CNN is two-dimensional, and its receptive field can extract local spatial features.

**Figure 4:**
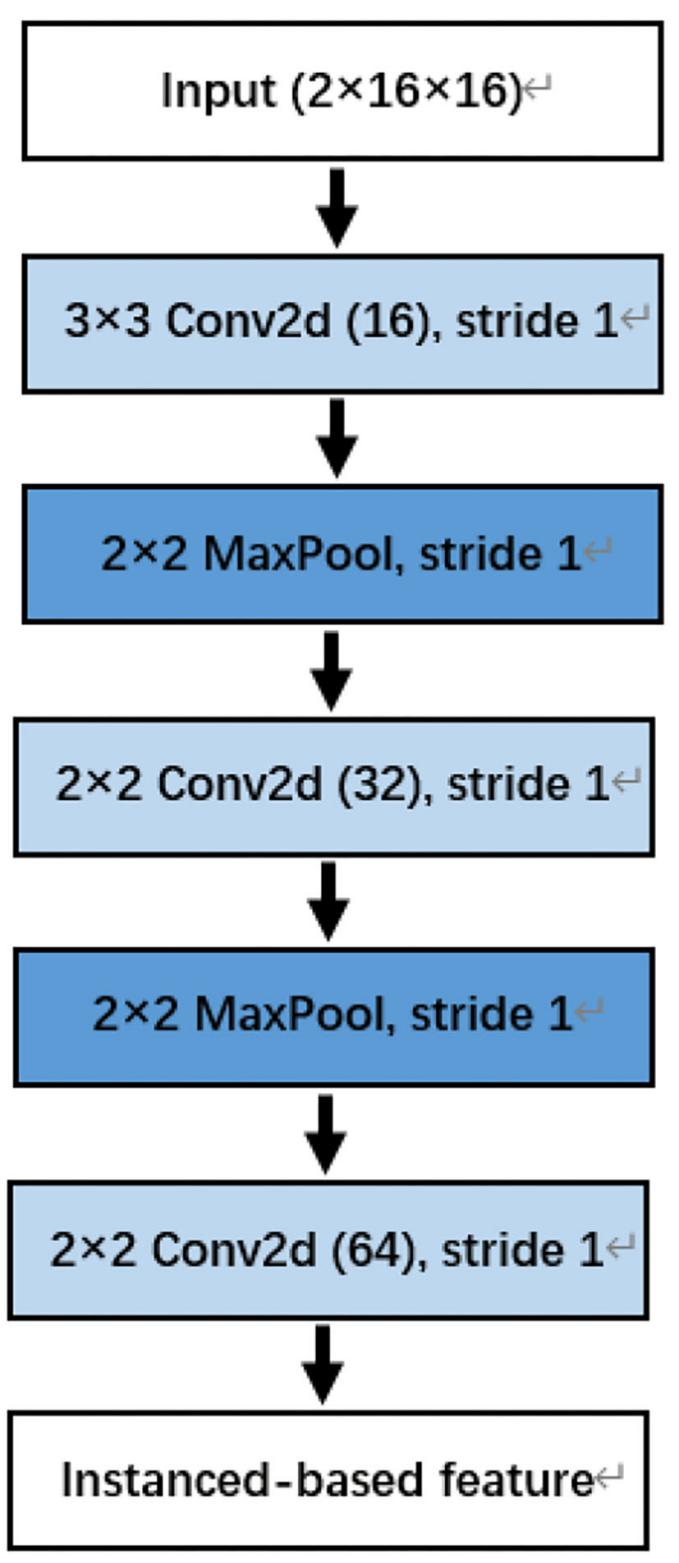
The convolutional neural network (CNN) for instanced-based feature extraction. Note that the last two dimensions of input are the number of channels. For example, the Dog_1 in the Kaggle dataset has 16 channels.

The convolutional neural network in this paper consists of three convolution blocks. Each convolution block contains convolution layer, batch normalization, and ReLU nonlinear activation function. Except for the last convolution block, each convolution block also contains a maximum pooling layer. The details are shown in Figure 4.

Through CNN, we have completed the extraction of spatial information, which we call an instance-based feature.

#### 3.2.2. Sequence-based feature detection and prediction

While extracting an instance-based feature, we used LSTM to extract a sequence-based feature simultaneously. To extract the sequence information of a segment, we did not to transform the segment. Given a segment with a size of (N, M), we regarded it as a time series with a length of M, and each element of the sequence is an N-dimensional vector. That is to say, the segment matrix is regarded as a time series composed of column vectors, which we can input it into LSTM to extract a sequence feature.

**Figure 5:**
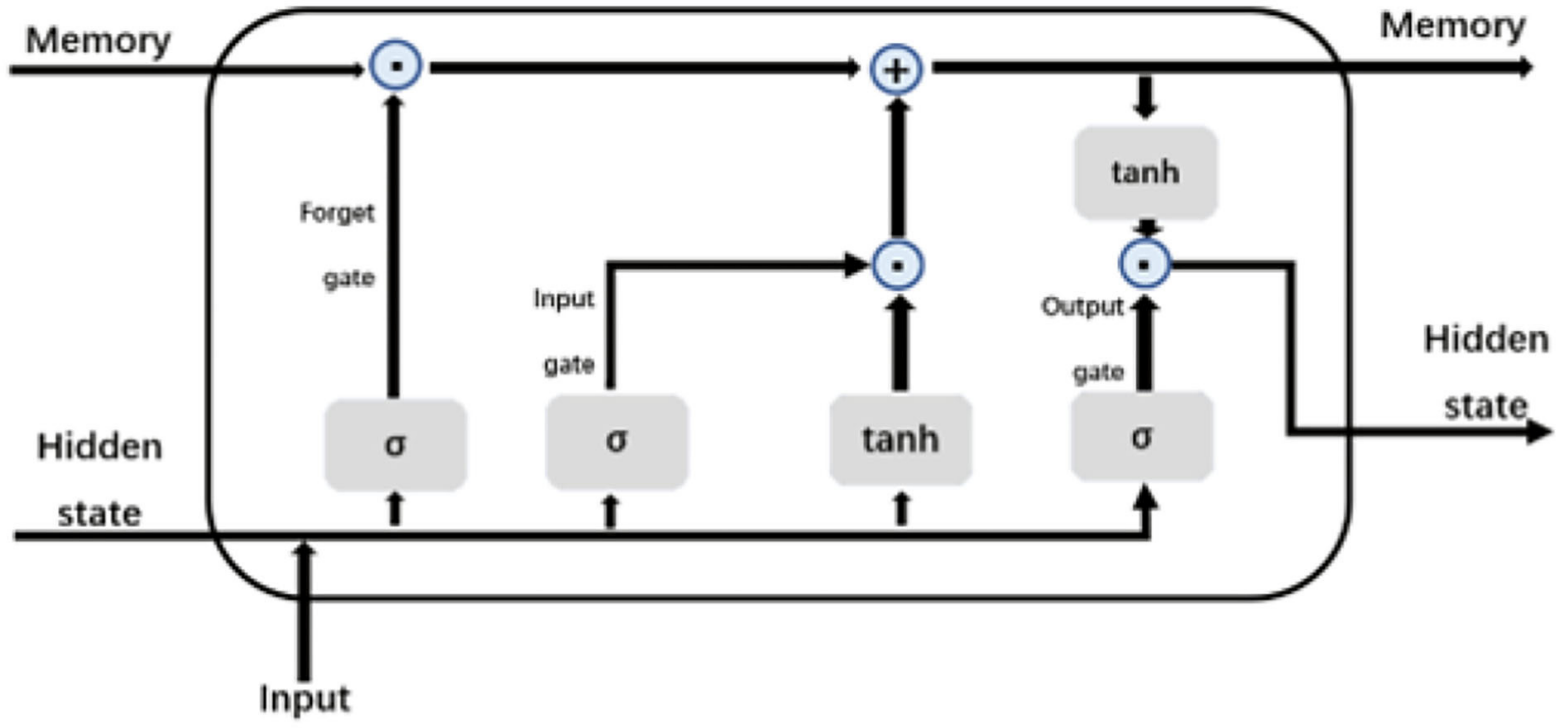
LSTM memory cell.

The difference between RNN and traditional multilayer perceptron is that it is a neural network with a hidden state. The hidden state can capture the historical information of the sequence up to the current time step, to extract the sequence information, and the number of parameters will not increase with the increase of the time step. However, the basic RNN is difficult to save the sequence information for a long time. One of the earliest methods to solve this problem is LSTM (Hochreiter and Schmidhuber, 1997). LSTM is a higher-order version of RNN, which can overcome many problems encountered during RNN training, such as gradient explosion and gradient vanishing. The LSTM contains memory cells. To control the memory cell, LSTM also includes input, forget, and output gates, which can decide when to remember or ignore the input in the hidden state through a special mechanism. The specific structure of the LSTM memory cell is shown in Figure 5. We use 256 LSTM memory cells in this model.

Through LSTM, we have completed the extraction of sequence information, which we call the sequence-based feature.

So far, we have obtained instance-based features using CNN and sequence-based features using LSTM. For the instance-based feature, we flat it into a vector and concatenate it with the sequence-based feature to obtain the final high-order feature.

The two sub-networks are followed by the three fully connected layers. The first and second fully connected layers were activated with ReLU nonlinear activation function, while the last used the SoftMax activation function to obtain the final probability distribution. The output sizes were 128, 64, and 2, respectively. Both of the fully connected layers have a dropout rate of 0.5. It is only used in the training stage, which can prevent overfitting and improve the robustness of the model.

#### 3.2.3. Loss function and hyperparameter setting

Since the model is a binary classification model, we use the cross-entropy loss function, and its formula is as follows, where y is the predicted output and ŷ is the desired output.


(2) $$\begin{array}{l} l\left(y,ŷ\right)=-\left[y\log\left(ŷ\right)+\left(1-y\right)\log\left(1-ŷ\right)\right] \end{array}$$

When training the model, this paper used the Adam algorithm to update the weight parameters, with the batch size set to 512, and the learning rate set to 0.0003. The proposed model is implemented in Python 3.8.11 using TensorFlow 2.3.1, with Ubuntu 18.04 operating system. The flowchart of our proposed model is shown in Figure 2.

## 4. Case studies

### 4.1. Data description

#### 4.1.1. Kaggle dataset

In this paper, the first dataset we used is the American Seizure Society Seizure Prediction Challenge (Kaggle) dataset (Brinkmann et al., 2016), which is publicly available. The dataset consists of a long-term iEEG of five canine subjects and two human subjects. It contains 50 seizures and 627.6 inter-ictal hours. For canine subjects, the EEG signals are sampled at a sampling rate of 400 Hz, recorded from 16 implanted electrodes for Dog_1 to Dog_4 and 15 electrodes for Dog_5. While for human subjects, the EEG signals are sampled at a sampling rate of 5,000 Hz, recorded from 15 implanted electrodes for Patient_1 and 24 implanted electrodes for Patient_2. To the influence of different kinds of subjects on the experimental results, we only selected canine subjects as the research objects. In addition, like most recent studies (Cheng et al., 2021; Gao et al., 2022; Yan et al., 2022), a period of 30 min before each seizure was defined as the prei-ctal period.

#### 4.1.2. CHB-MIT scalp EEG dataset

This dataset (Shoeb, 2009) was collected at the Boston Children's Hospital, consisting of sEEG recordings from pediatric subjects with intractable seizures. It consisted of 24 cases from 23 subjects, as well as the gender and age data of each subject. Each case contains 9–42 consecutive EDF files, most of which record sEEG signals with a duration of 1 h. All signals are sampled with 16-bit resolution at a sampling frequency of 256 Hz. The International 10–20 system of EEG electrode positions and nomenclature are used for these recordings. The annotation file records the specific time information of the beginning and end of each seizure. Because the dataset does not specifically distinguish between inter-ictal and pre-ictal episodes, this paper follows the labeling method of the Kaggle dataset. In addition, due to the limitation of hardware, some files have the problem of inconsistent channels.

To avoid the heterogeneity of data, we selected 18 electrodes that are included in most EEG signals: P8-O2, F8-T8, F7-T7, P7-O1, FZ-CZ, FP1-F7, FP2-F8, T8-P8, F3-C3, C4-P4, CZ-PZ, T7-P7, F4-C4, C3-P3, P3-O1, FP2-F4, FP1-F3, and P4-O2. We treated seizures with intervals of less than 30 min as the same seizure, and we required cases to have at least three seizures and sufficient inter-ictal data. Under the constraints of the above conditions, we selected a total of 18 cases, including 84 seizures.

### 4.2. Model evaluation

In this paper, we used a Leave-one-out cross to ensure the robustness and generalization ability of the proposed model. Specifically, given a data of a subject, if it has N pre-ictal data, one was considered as the test set, and the remaining N-1 as the training set and validation set. In addition, the ratio of the training set and validation set is 80%:20%. The same processing method is used for inter-ictal data. After that, the model is trained on the N-1 inter-ictal and pre-ictal data, and the remaining one is tested. The process is then repeated by changing the pre-ictal data under test, which can cover all the pre-ictal data and the tested pre-ictal data is unseen during the training. After the N experiments, the mean value is taken to get the final result.

Performance measures used in this work are based on the analysis of true positives (TP), true negatives (TN), false positives (FP), and false negatives (FN) instances classified during the testing phase. When evaluating the model on the test set, such as the general binary classification problem, we calculated the Acc(accuracy), Sen(sensitivity), Spe(specificity), and F1-score which are defined as follows:


(3) $$\begin{array}{l} \text{accuracy}=\frac{TP+TN}{TP+TN+FP+FN} \end{array}$$

(4) $$\begin{array}{l} \text{sensitivity}=\text{recall}=\frac{TP}{TP+FN} \end{array}$$

(5) $$\begin{array}{l} \text{specificity}=\frac{TN}{TN+FP} \end{array}$$

(6) $$\begin{array}{l} \text{precision}=\frac{TP}{TP+FP} \end{array}$$

(7) $$\begin{array}{l} \text{F}1-\text{score}=\frac{2\times \text{recall}\times \text{precision}}{\text{recall}+\text{precision}} \end{array}$$

In addition to Acc, Sen, Spe, and F1-score, we also calculated area under the curve (AUC) for model evaluation.

## 5. Results

In this section, we evaluated the model with two datasets: the Kaggle dataset and the CHB-MIT dataset. We first uniformly resampled all EEG signals at 256 Hz. Then, we performed ablation experiment to compare the performance of three models: CNN, LSTM, and multi-frame network to verify that our proposed multi-frame model is better than the model of a single frame. Since the model we proposed is patient-specific, for each dataset, we evaluated the model for the specific patient of the dataset.

Table 1 summarizes the experimental results of CNN and multi-frame network on the Kaggle dataset. We observed that the performance of the multi-frame network is better than CNN in terms of mean accuracy, sensitivity, and specificity. This is because CNN extracts instance-based features, while multi-frame network extracts instance-based features and sequence-based features simultaneously, which improves the average values of accuracy, sensitivity, and specificity by 1.42, 0.01, and 2.81%, respectively. Table 2 summarizes the results of LSTM and multi-frame network on the Kaggle dataset. Similarly, compared with LSTM, the mean accuracy, sensitivity, and specificity of the multi-frame network are improved by 10.99, 17.49, and 4.44%, respectively. Therefore, from the average of the results of all subjects, the multi-frame network is better than the single-frame network in the classification of inter-ictal and pre-ictal episodes based on EEG.

**Table 1:** Results achieved in the Kaggle dataset using the convolutional neural network (CNN) and the multi-frame network.

**Subject**	**No.Sei^a^**	**CNN**			**multi-frame**		
		**(instance-based)**			**network**		
		**Acc**	**Sen**	**Spe**	**Acc**	**Sen**	**Spe**
		**(*%*)**	**(*%*)**	**(*%*)**	**(*%*)**	**(*%*)**	**(*%*)**
Dog_1	4	63.05	60.18	65.91	67.13	60.94	73.31
Dog_2	7	73.34	75.87	70.82	73.40	74.11	72.70
Dog_3	12	59.29	70.83	47.77	59.56	64.77	54.35
Dog_4	17	59.58	56.98	62.31	60.86	58.64	63.19
Dog_5	5	75.10	76.01	74.19	76.49	81.50	71.50
Mean		66.07	67.98	64.20	67.49	67.99	67.01

^a^Number of seizures.

**Table 2:** Results achieved in the Kaggle dataset using LSTM and the multi-frame network.

**Subject**	**No.Sei^a^**	**CNN**			**multi-frame**		
		**(instance-based)**			**network**		
		**Acc**	**Sen**	**Spe**	**Acc**	**Sen**	**Spe**
		**(*%*)**	**(*%*)**	**(*%*)**	**(*%*)**	**(*%*)**	**(*%*)**
Dog_1	4	62.07	54.24	69.89	67.13	60.94	73.31
Dog_2	7	55.46	48.08	62.84	73.40	74.11	72.70
Dog_3	12	52.04	44.79	59.28	59.56	64.77	54.35
Dog_4	17	51.84	44.78	59.25	60.86	58.64	63.19
Dog_5	5	61.11	60.63	61.58	76.49	81.50	71.50
Mean		56.50	50.50	62.57	67.49	67.99	67.01

^a^Number of seizures.

**Figure 6:**
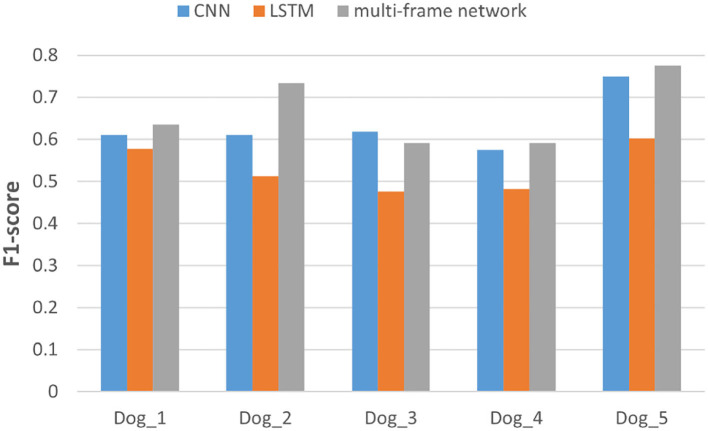
F1-score analysis of the three models for subjects in the Kaggle dataset.

**Figure 7:**
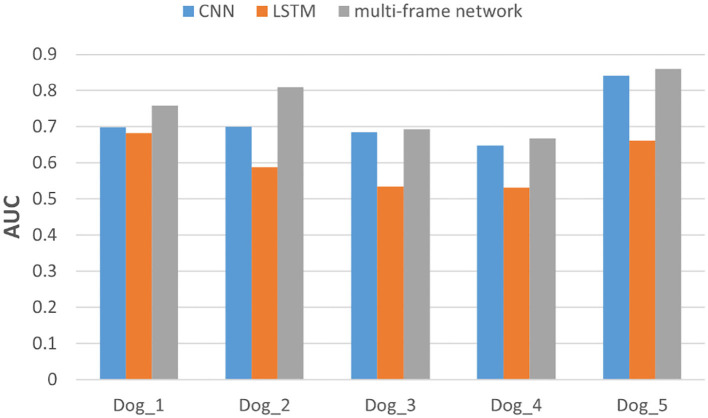
The area under the curve (AUC) analysis of the three models for subjects in Kaggle dataset.

In addition, we also evaluated the performance of each subject in the dataset. Specifically, we compared the F1-score and AUC of CNN, LSTM, and multi frame networks on each subject's data. The F1-score and AUC analysis are illustrated in Figures 6, 7, respectively. We found that the F1-score and AUC of the multi-frame network were higher than those of the single-frame network except for Dog_3 whose F1-score of the multi-frame network is slightly lower than that of its CNN. It clearly illustrates the advantages of the multi-frame network over the single-frame network. Higher F1-score and AUC showed that the proposed model in this paper is more stable and robust. For most patients, the prediction ability of the multi-frame network is better than that of the single-frame network.

**Figure 8:**
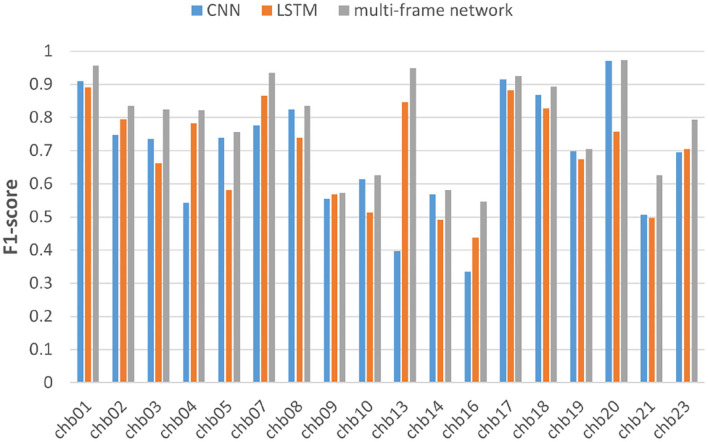
F1-score analysis of the three models for subjects in the CHBMIT dataset.

**Figure 9:**
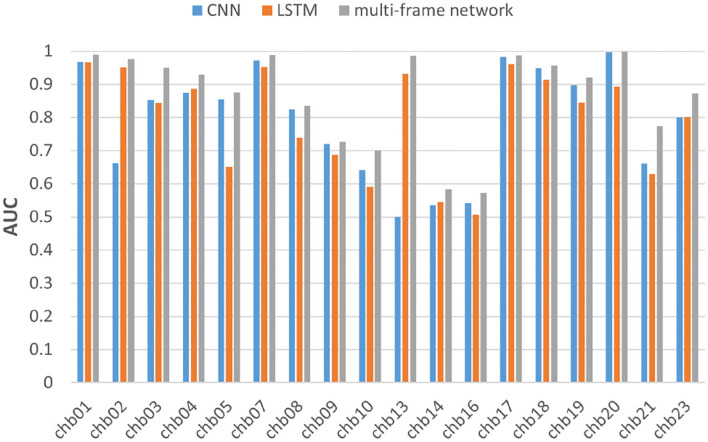
AUC analysis of the three models for subjects in the CHBMIT dataset.

We also performed the same experiment on the CHB-MIT dataset. Table 3 summarizes the experimental results of CNN and multi-frame network on the CHB-MIT dataset. Table 4 summarizes the experimental results of LSTM and multi-frame network on the CHB-MIT dataset. F1-score and AUC analysis for each subject are illustrated in Figures 8, 9, respectively. We observed that the accuracy, sensitivity, and specificity of the multi-frame network were improved by 9.42, 3.72, and 15.05%, respectively, compared with CNN, and 7.76, 10.01, and 5.58%, respectively, compared with LSTM. In addition, for each case, the F1-score and AUC of the multi-frame network were higher than that of the single-frame network, thus, our proposed framework still has advantages.

**Table 3:** Results achieved in the CHB-MIT dataset using CNN and the multi-frame network.

**Subject**	**No.Sei^a^**	**CNN**			**multi-frame**		
		**(instance-based)**			**network**		
		**Acc**	**Sen**	**Spe**	**Acc**	**Sen**	**Spe**
		**(*%*)**	**(*%*)**	**(*%*)**	**(*%*)**	**(*%*)**	**(*%*)**
chb01	7	91.29	91.86	90.71	96.24	95.34	97.14
chb02	3	64.28	96.22	32.33	88.13	78.26	97.99
chb03	6	72.30	82.18	62.43	85.81	79.29	92.33
chb04	3	62.53	58.91	66.16	78.73	89.13	68.32
chb05	5	76.08	70.66	81.50	77.77	72.53	83.00
chb07	3	68.07	99.54	36.59	94.34	91.00	97.68
chb08	5	77.01	79.44	73.97	77.89	82.04	72.70
chb09	4	65.34	48.00	82.68	65.84	51.01	80.67
chb10	6	57.96	71.84	44.08	64.54	61.90	67.19
chb13	5	50.00	60.00	40.00	95.24	94.91	95.57
chb14	5	54.20	62.09	46.32	56.89	60.59	53.18
chb16	6	52.97	45.36	60.57	57.36	54.89	59.84
chb17	3	92.41	88.19	96.64	93.35	89.00	97.70
chb18	6	87.70	85.10	90.30	89.90	89.56	90.25
chb19	3	76.29	61.09	91.49	77.44	61.98	92.89
chb20	5	97.53	97.53	97.52	97.82	97.47	98.17
chb21	4	60.84	41.97	79.70	67.43	58.13	76.74
chb23	5	80.96	97.70	65.43	92.63	97.69	87.94
Mean		71.54	74.32	68.80	80.96	78.04	83.85

^a^Number of seizures.

**Table 4:** Results achieved in the CHB-MIT dataset using LSTM and the multi-frame network.

**Subject**	**No.Sei^a^**	**CNN**			**multi-frame**		
		**(instance-based)**			**network**		
		**Acc**	**Sen**	**Spe**	**Acc**	**Sen**	**Spe**
		**(*%*)**	**(*%*)**	**(*%*)**	**(*%*)**	**(*%*)**	**(*%*)**
chb01	7	89.90	86.82	92.99	96.24	95.34	97.14
chb02	3	84.22	73.09	95.34	88.13	78.26	97.99
chb03	6	74.70	61.04	88.36	85.81	79.29	92.33
chb04	3	76.00	81.39	70.61	78.73	89.13	68.32
chb05	5	60.80	55.90	65.69	77.77	72.53	83.00
chb07	3	87.98	83.43	92.53	94.34	91.00	97.68
chb08	5	69.29	78.45	57.87	77.89	82.04	72.70
chb09	4	59.94	54.47	65.41	65.84	51.01	80.67
chb10	6	55.99	47.80	64.18	64.54	61.90	67.19
chb13	5	85.58	81.94	89.22	95.24	94.91	95.57
chb14	5	53.24	46.18	60.31	56.89	60.59	53.18
chb16	6	50.19	41.64	58.74	57.36	54.89	59.84
chb17	3	89.21	86.04	92.39	93.35	89.00	97.70
chb18	6	83.19	84.34	82.05	89.90	89.56	90.25
chb19	3	73.10	57.52	88.69	77.44	61.98	92.89
chb20	5	78.78	68.51	89.04	97.82	97.47	98.17
chb21	4	57.83	43.45	72.21	67.43	58.13	76.74
chb23	5	87.68	92.50	83.20	92.63	97.69	87.94
Mean		73.20	68.03	78.27	80.96	78.04	83.85

^a^Number of seizures.

It should be noted that in the CHBMIT dataset (sEEG), the sampling frequency is 256 Hz, and in the Kaggle dataset (iEEG), the sampling frequency of canine subjects is 500 Hz. Since we uniformly downsampled to 256 Hz in the experiment, some information may be lost. This led to experimental results on the Kaggle dataset that were not as good as the CHBMIT dataset, but the experimental results still showed that the performance of the multi-frame network was better than that of the single-frame network. Therefore, through experiments on the Kaggle dataset and the CHB-MIT dataset, respectively, we concluded that whether on sEEG or iEEG, a multi-frame network is always superior to the single-frame network.

## 6. Discussion

In this paper, a model using the multi-frame network is proposed to predict seizure episodes because it has the advantage of extracting instance-based features and sequence-based features simultaneously. To further evaluate the effectiveness of our model, we compared it with CNN-LSTM and conducted experiments on the same datasets. The difference between the two models was the feature extraction methodology, where the CNN-LSTM first used CNN to extract instance-based features, and then used LSTM to extract sequence-based features. For each dataset, the mean accuracy, sensitivity, specificity, F1-score, and AUC of all subjects were utilized to test the final experimental results.

**Figure 10:**
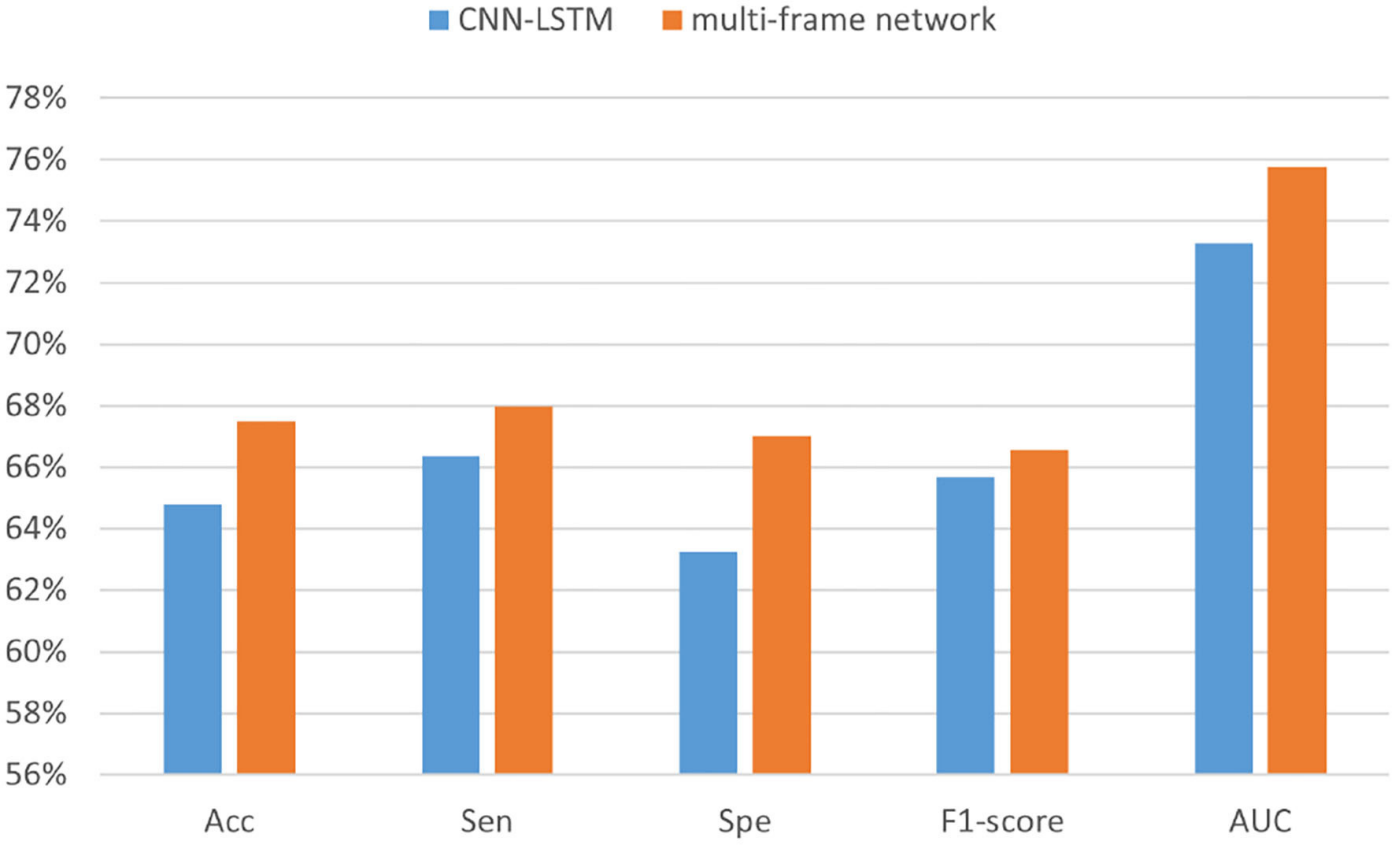
The performance analysis of CNN-LSTM and the multi-frame network in the Kaggle dataset.

**Figure 11:**
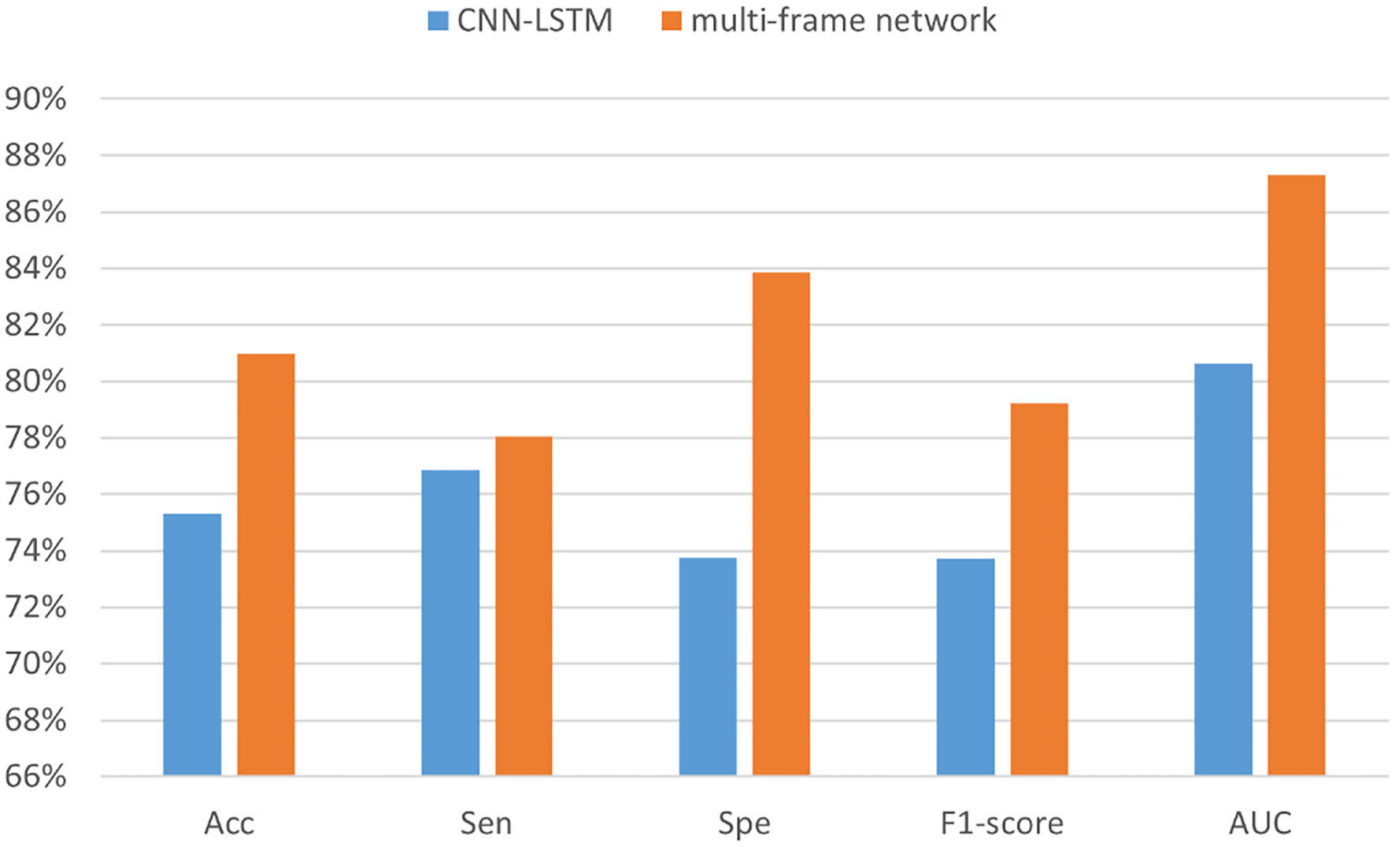
The performance analysis of CNN-LSTM and the multi-frame network in the CHB-MIT dataset.

Table 5 summarizes the experimental results of the two networks on the Kaggle dataset. We observed that according to all performance measures, the multi-frame network was higher than CNN-LSTM. In addition, Figure 10 shows that the reason for accuracy improvement mainly comes from the optimization of specificity, and it is an important index to measure the discriminative of the model for inter-ictal. It can reduce the false alarm rate of epilepsy prediction model, which has important practical significance. Moreover, Figure 11 and Table 6 are the experimental results on the CHB-MIT dataset, which also show the advantages of our proposed approach.

**Table 5:** CNN-LSTM vs. multi-frame network in the Kaggle dataset.

**Model architecture**	**Acc (*%*)**	**Sen (*%*)**	**Spe (*%*)**	**F1-score**	**AUC**
CNN-LSTM	64.79	66.36	63.26	0.66	0.73
multi-frame network	67.49	67.99	67.01	0.67	0.76

**Table 6:** CNN-LSTM vs. multi-frame network in the CHB-MIT dataset.

**Model architecture**	**Acc (*%*)**	**Sen (*%*)**	**Spe (*%*)**	**F1-score**	**AUC**
CNN-LSTM	75.32	76.87	73.76	0.74	0.81
multi-frame network	80.96	78.04	83.85	0.80	0.87

According to the experimental results, we found that the features extracted by the multi-frame network were more discriminative than those extracted by CNN-LSTM, especially in the inter-ictal. Analyzing the reasons for the experimental results, it can be concluded that CNN-LSTM first extracts instance-based features and then extracts sequence-based features, which may result in the loss of some information. Therefore, it can be improved by using multi-frame network to extract features simultaneously.

**Figure 12:**
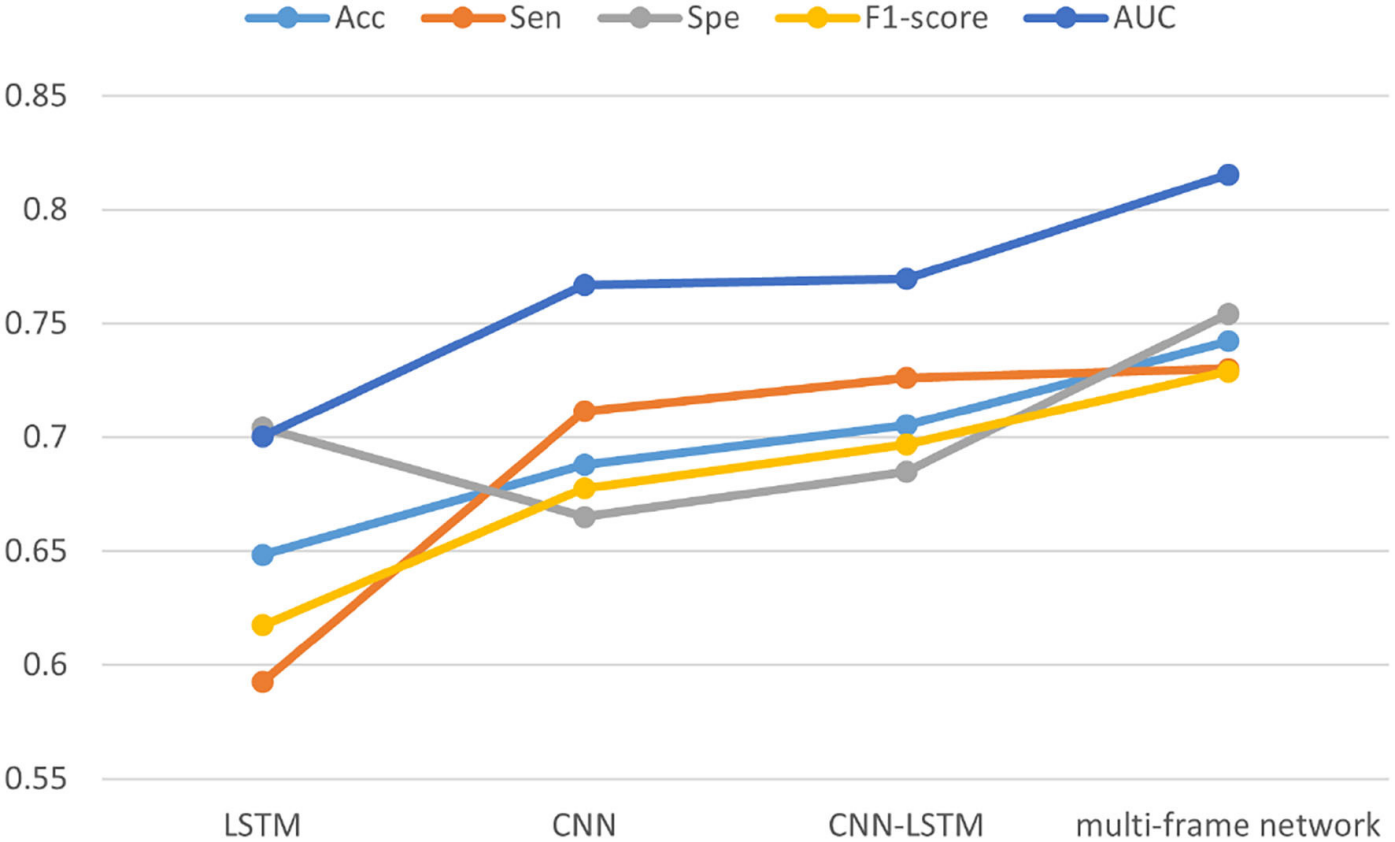
Comprehensive performance analysis of the four models in two datasets.

Finally, we summarized all the experimental results, and Figure 12 shows the numerical ones. In the figure, each point represents the mean value of the corresponding performance measure of different networks on the two datasets. We concluded that with the continuous optimization of network architecture, accuracy, sensitivity, specificity, F1-score, and AUC of the model were also gradually improved. Specifically, compared with LSTM, CNN, and CNN-LSTM, the accuracy of the multi-frame network increased over 9.38, 5.42, and 3.68% respectively, the sensitivity was improved by 13.75, 1.87, and 0.40%, respectively, the specificity was improved by 5.01, 8.93, and 6.92%, respectively, the F1-score was improved by 0.11, 0.05, and 0.03, respectively, and the AUC was improved by 0.12, 0.05, and 0.05, respectively.

## 7. Conclusions and future work

The seizure prediction model has an important practical significance to improve the quality of life of patients with epilepsy. Most of the existing models are based on the traditional methods, such as CNN or RNN, and the framework is relatively not easy to be improved and generalized for further research on prediction accuracy. In this paper, we proposed a multi-frame network to extract instance-based and sequence-based features simultaneously to get discriminative high-order features. Based on the results from the ablation analysis, the effectiveness of the multi-frame network is validated and compared with a single-frame network, which provides a novel and interesting research idea in designing the seizure prediction model. Most importantly, we verified that the model proposed in this paper can not only obtain the most discriminant features but also identify inter-ictal episodes more effectively, which is of great practical significance. In addition, the model demonstrated good generalization and the experiments on iEEG represented by the Kaggle dataset and sEEG represented by the CHB-MIT dataset, which is superior to existing methods.

Since this paper aimed to study the architecture of the model, the experiments were conducted only based on the basic CNN and LSTM models, and some novel and advanced frames or their variants were not considered. However, researchers can change these frames by combining them according to the novel idea we proposed, which is also considered one of our future works.

## Data availability statement

Publicly available datasets were analyzed in this study. This data can be found here: https://www.kaggle.com/competitions/seizure-prediction/data (Kaggle); https://physionet.org/content/chbmit/1.0.0/ (CHBMIT).

## Author contributions

LL and FZ: model building, analysis, and experiments. YW, SM, XZ, and GN: related work, proofreading, and experiments. All authors contributed to the article and approved the submitted version.

## Funding

This research was supported by the Natural Science Foundation of Tianjin City (Tianjin University Medical and Industrial Foundation). The authors appreciate Tianjin University Medical and Industrial Foundation for financial support under Grant No. 20JCZDJC00810.

## Conflict of interest

The authors declare that the research was conducted in the absence of any commercial or financial relationships that could be construed as a potential conflict of interest.

## Publisher's note

All claims expressed in this article are solely those of the authors and do not necessarily represent those of their affiliated organizations, or those of the publisher, the editors and the reviewers. Any product that may be evaluated in this article, or claim that may be made by its manufacturer, is not guaranteed or endorsed by the publisher.
